# Complex Patterns of Human Antisera Reactivity to Novel 2009 H1N1 and Historical H1N1 Influenza Strains

**DOI:** 10.1371/journal.pone.0039435

**Published:** 2012-07-17

**Authors:** Donald M. Carter, Hai-Rong Lu, Chalise E. Bloom, Corey J. Crevar, Joshua L. Cherry, David J. Lipman, Ted M. Ross

**Affiliations:** 1 Center for Vaccine Research, School of Medicine, University of Pittsburgh, Pittsburgh, Pennsylvania, United States of America; 2 Graduate Program in Immunology, School of Medicine, University of Pittsburgh, Pittsburgh, Pennsylvania, United States of America; 3 Department of Microbiology and Molecular Genetics, School of Medicine, University of Pittsburgh, Pittsburgh, Pennsylvania, United States of America; 4 National Center for Biotechnology Information, National Library of Medicine, National Institutes of Health, Bethesda, Maryland, United States of America; The University of Adelaide, Australia

## Abstract

**Background:**

During the 2009 influenza pandemic, individuals over the age of 60 had the lowest incidence of infection with approximately 25% of these people having pre-existing, cross-reactive antibodies to novel 2009 H1N1 influenza isolates. It was proposed that older people had pre-existing antibodies induced by previous 1918-like virus infection(s) that cross-reacted to novel H1N1 strains.

**Methodology/Principal Findings:**

Using antisera collected from a cohort of individuals collected before the second wave of novel H1N1 infections, only a minority of individuals with 1918 influenza specific antibodies also demonstrated hemagglutination-inhibition activity against the novel H1N1 influenza. In this study, we examined human antisera collected from individuals that ranged between the ages of 1 month and 90 years to determine the profile of seropositive influenza immunity to viruses representing H1N1 antigenic eras over the past 100 years. Even though HAI titers to novel 2009 H1N1 and the 1918 H1N1 influenza viruses were positively associated, the association was far from perfect, particularly for the older and younger age groups.

**Conclusions/Significance:**

Therefore, there may be a complex set of immune responses that are retained in people infected with seasonal H1N1 that can contribute to the reduced rates of H1N1 influenza infection in older populations.

## Introduction

The influenza antigens hemagglutinin (HA) and neuraminidase (NA) are the major surface glycoproteins of the virus and thus immune protective targets. Changes (antigenic drift and shift) in these HA and NA proteins can result in evasion of pre-existing neutralizing antibodies within a host. Antigenic shifts led to 3 influenza pandemics over the last century resulting in significant morbidity and mortality. The 1918 pandemic was the most severe, killing up to 50 million people worldwide. The 1918 influenza virus was recently reconstructed from preserved patient specimens [1], [2], [3] and is similar in sequence to the swine H1N1 viruses from that era [1]. Human H1N1 serotypes persisted as seasonal influenza until 1957, when they were replaced by the H2N2 virus [4]. In 1968, the H2N2 isolates were replaced in the human population by viruses of the H3N2 subtype. In 1977, the H1N1 virus reappeared in human populations. Since then, H1N1 and H3N2 influenza have been circulating together with influenza B viruses among humans.

In April 2009, the first cases of novel influenza H1N1 were identified in North America. Our group and others demonstrated that of the ∼65 million people that were infected in the United States by the end of 2009, infection and disease were highest in school-age children, and severe cases were underrepresented in elderly adults [5], [6], [7], [8], [9]. Structural analysis of the HA shows a conservation within antigenic regions of 1918 and 2009 pandemic HA proteins that is not present in contemporary seasonal H1N1 viruses [10], [11]. Antigenic similarities, together with the abnormal protection from severe disease in the elderly population, led to the hypothesis that exposure to 1918-like viruses confers cross-protective immune responses to novel H1N1 isolates [12], [13]. Several studies have indicated cross-reactive antibodies to the 2009 pandemic H1N1 viruses in elderly human populations [14] with monoclonal antibodies derived from survivors of the 1918 pandemic able to cross-neutralize 2009 pandemic viruses [15]. Additionally, direct evidence of the cross-protective efficacy elicited by exposure to 1918-like viruses has been demonstrated in small animal models [16], [17]. Therefore, the view emerged that the 2009 HA differed little from its 1918 ancestor with respect to the antibody responses, and that exposure to seasonal H1N1 in the early twentieth century could explain the observed protection of older adults from the 2009 pandemic.

However, serological data collected between 2009 and 2011 shows that only a minority of individuals with 1918 influenza-specific antibodies also recognized the novel H1N1 influenza [9]. Our group examined human sera from individuals ranging between 1 month and 90 years of age [9]. Although antibody reactivity toward the novel 2009 H1N1 viruses and the 1918 influenza viruses are correlated, this correlation is not extraordinarily strong. Furthermore, the age-dependences of particular antibody reactivity and their relationships to each other are not readily explained by simple models. These results do not support the notion that the novel 2009 H1N1 influenza viruses are nearly antigenically equivalent to the 1918 influenza viruses and suggest a complex relationship between a life-long history of infection and the resulting antibody profile. These results presented in this report also have implications for pre-pandemic vaccine priming for emerging influenza subtypes.

## Results

### Antibodies to Novel H1N1 Influenza

**Figure 1:**
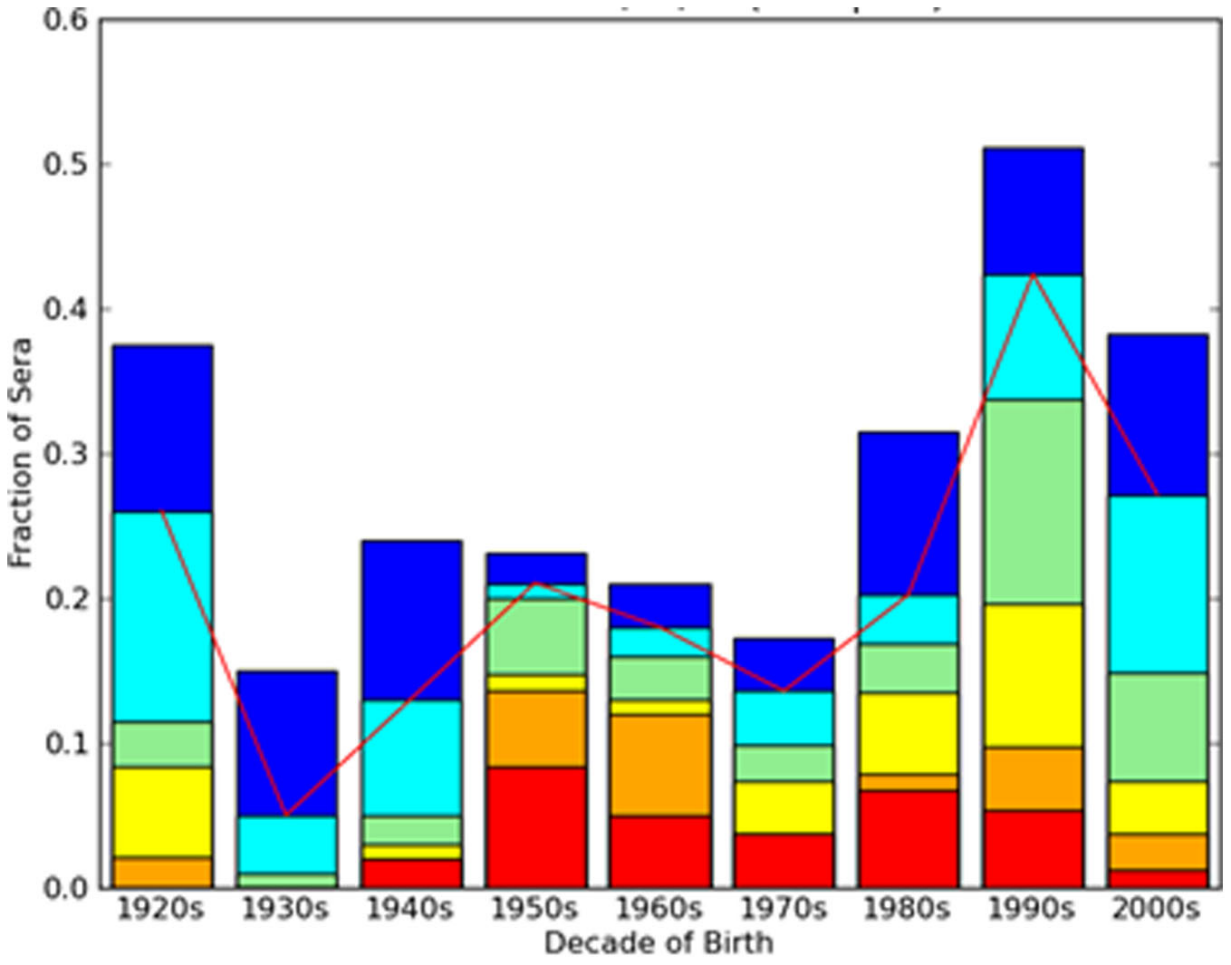
Fraction of sera positive for A/Mexico/4108/2009 (novel H1N1) influenza by HAI titer by decade of birth. The number of serum samples was categorized by HAI titer per age group. Dark blue = 1∶20; Light blue = 1∶40; Green = 1∶80; Yellow = 1∶160; Orange = 1∶320; Red = 1∶640.

In late November, 2009, approximately 2–4 weeks after the peak of the fall wave in Allegheny County, Pennsylvania, serum samples were collected anonymously from 846 persons that ranged in age from 1 month to 90 years of age [14]. As previously described, the HAI titer was determined for each serum sample collected (≥1∶40 HAI titer as positive) for the novel H1N1 isolate A/California/7/2009 [9] or A/Mexico/4108/2009 (Fig. 1). The percentage of HAI positive samples was highest for the very young (10–19 year-olds and 0–9 year-olds) and very old (80–90 year-olds). The test for trend demonstrated increasing seropositivity for novel H1N1 among younger cohorts (p  = 0.001). Examination of sera with HAI high titers ≥1∶320 reveals that the fraction of individuals with high titers (orange and red regions in Fig. 1) is highest among people born in the 1950s and 1960s and lowest among the oldest age groups (1920s and 1930s).

### 1918 Influenza Antibody Cross-reactivity with Novel H1N1 Influenza

**Figure 2:**
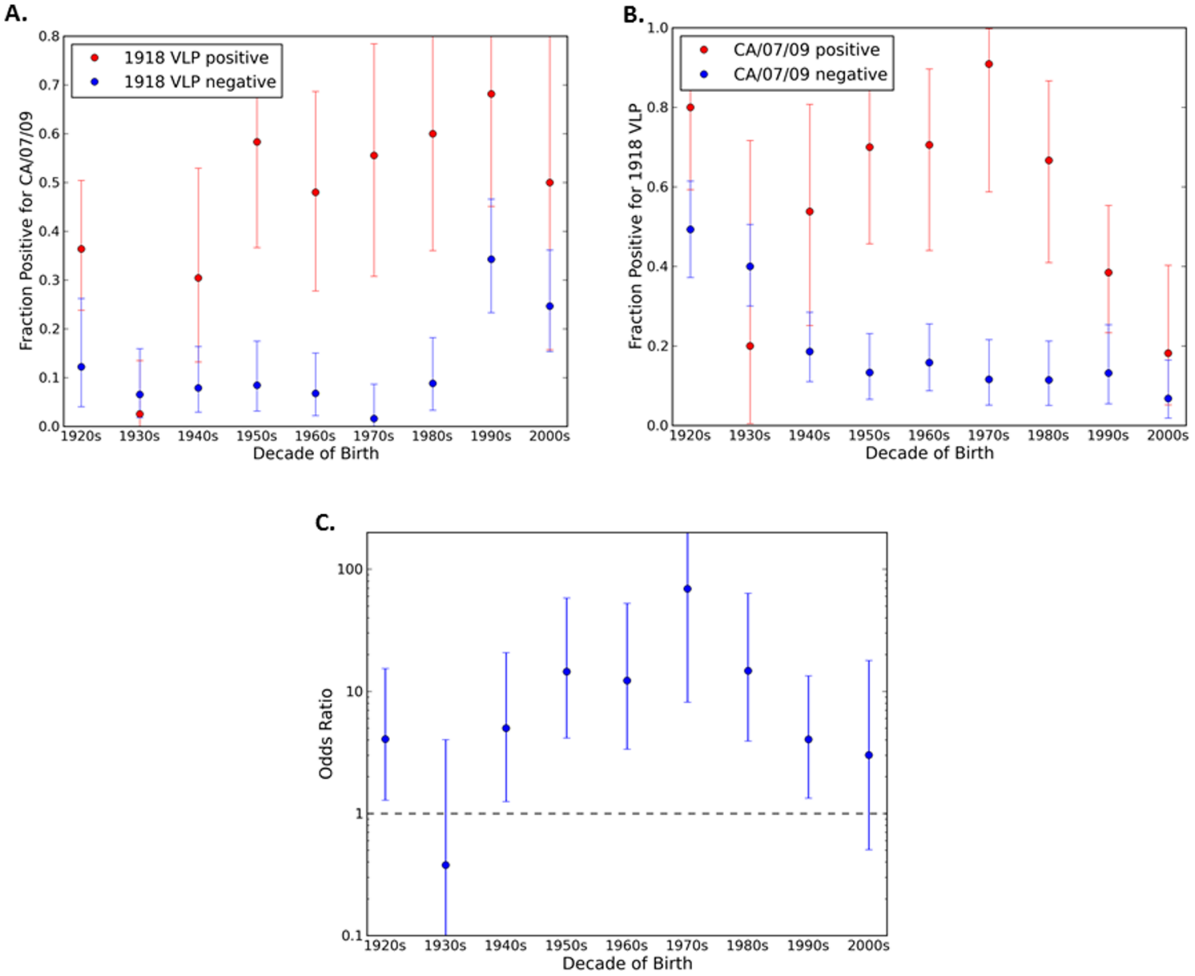
Seropositivity (HAI≥1∶40) for both novel H1N1 and 1918 influenza by decade of birth. Panel (A): The percentage of serum samples positive for A/California/07/2009 (novel H1N1) among those that were positive (red) or negative (blue) for 1918 influenza. Panel (B): The percentage of serum samples positive for 1918 influenza among those that were positive (red) or negative (blue) for A/California/07/2009 (novel H1N1) influenza. Panel (C): Odds ratio for seropositivity for the two antigens. In all three panels the error bars indicate 95% confidence intervals.

If the novel 2009 virus and the 1918 virus are antigenically very similar, as required by some explanations of pre-existing immunity to the 2009 virus, we would expect a strong association between serum positivity for one and positivity for the other. As previously described [9], HAI titers (≥1∶40 HAI titer as positive) were determined for 1918 and novel H1N1 viruses for each serum sample collected from a cohort of people representing ages 1 month to 90 years of age for the novel H1N1 isolate A/California/7/2009 [9] or A/Mexico/4108/2009 (Fig. 1). The relationship between positivity for a novel H1N1 and for the 1918 virus (A/South Carolina/1/1918) is presented in Fig. 2. The odds ratio is greater than 1 (with p<0.05) for all birth decades other than the 1930s and 2000s (Fig. 2C), indicating that sera positive for one virus are more likely to be positive for the other. Nonetheless, only a fraction of the people with HAI antibodies against 1918 also had antibodies that recognized novel H1N1 and vice versa. For example, in sera collected from individuals born in the 1920s that recognized the 1918 virus, only 38% of the sera also recognized novel H1N1 (Fig. 2A). Although the two viruses are clearly related, many serum samples are positive for one virus, but negative for the other across all age groups. For the older cohorts, more than half of those positive for the 1918 virus are negative for the 2009 virus, suggesting that infection with 1918-like influenza need not elicit antibodies that react with the 2009 virus. The association between HAI positive titers is strongest for middle aged groups, whose earliest H1N1 exposure would likely have been to a virus (or vaccine) quite different from the 1918-like viruses.

### Hemagglutinin-inhibition Antibody Profiles Against H1N1 Influenza

**Figure 3:**
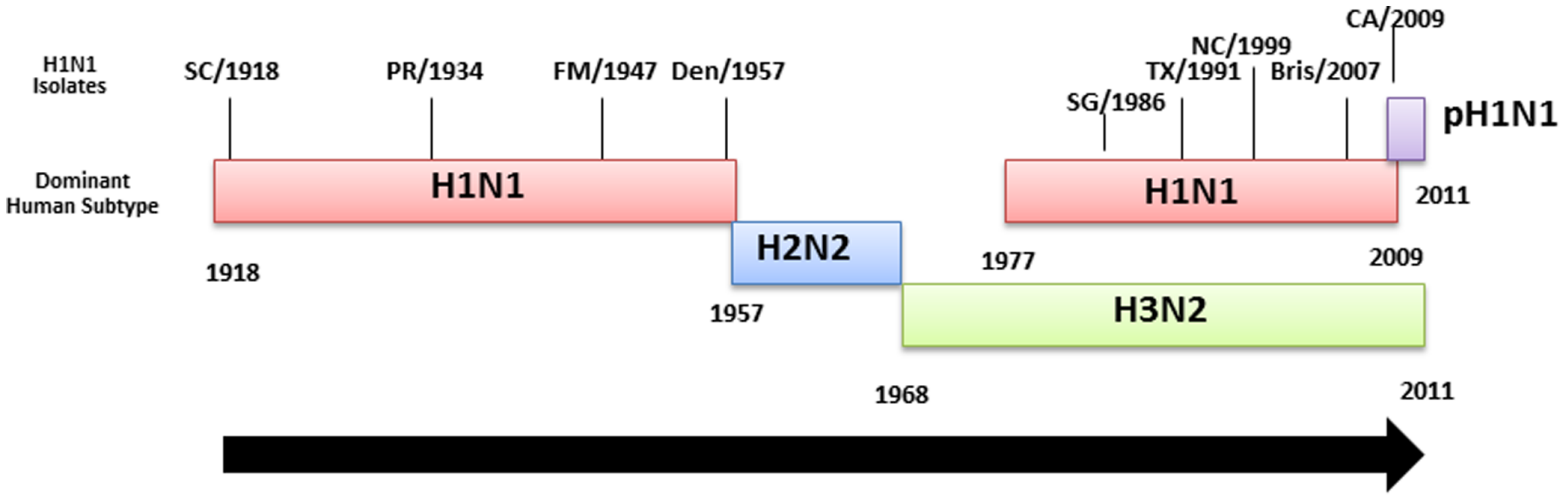
Schematic representation of influenza A subtypes circulating in the human population since 1918. Each subtype (colored boxes) and specific H1N1 strains used for analysis in this study are depicted chronologically.

These and other results suggest that an individual’s history of exposure to influenza viruses bears a complex relationship to protection from novel viruses. To explore this relationship, we tested each serum sample for HAI activity against a panel of human H1N1 influenza strains isolated during the past 100 years in the context of the history of human influenza infections since 1918 (Fig. 3). Previously, our group tested this cohort for seroprevalence against two seasonal H1N1 viruses [9] and included those results in an expanded panel seasonal H1N1 isolates for analysis. Four of the H1N1 strains were isolated between 1918 and 1957 and represent historical strains, whereas four of the strains were isolated between 1986 and 2007 and represent contemporary strains after the reintroduction of an H1N1 that is co-circulating with H3N2, but before the novel 2009 H1N1 pandemic.

**Figure 4:**
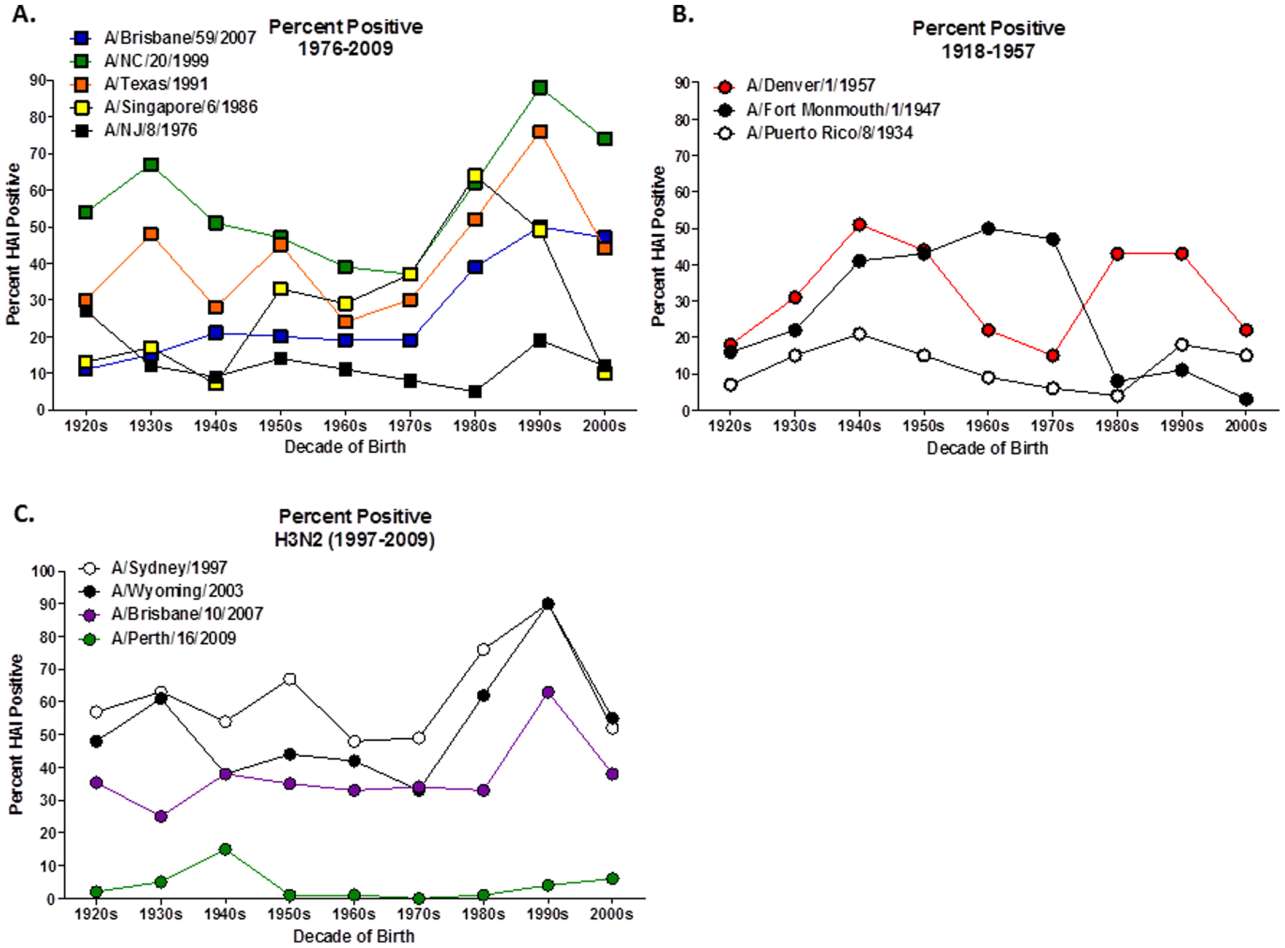
Seropositive samples for influenza A strains. The percentage of serum samples that were HAI positive per decade of birth were identified for (A) historical Human H1N1 strains; 1934–1957, (B) contemporary Human H1N1 strains; 1986–2007, (C) Human H3N2 strains.

As indicated previously [14], high HAI activity against A/South Carolina/1/1918 was rare except in the sample sets from the 1920s and 1930s. Sera with HAI activity against seasonal isolates from 1991 to 2007 were generally most prominent in the youngest (1990s and 2000s) cohorts (Fig. 4A). HAI activity against the 1986 virus was highest for those born in the 1980s and quite low for those born in the 2000s. These observations are broadly compatible with a simple model in which HAI activity toward a virus reflects exposure to a fairly closely related virus.

Other aspects of HAI activity against the H1N1 virus panel are not so simply explained. Few samples collected from any age group recognized A/Puerto Rico/8/1934. HAI activity against A/Fort Monmouth/1/1947 was low in those born in the 1980s and later. Interestingly however, sera collected from those born in the 1960s and 1970s have high titers to the 1947 isolate, while these same individuals generally have low HAI activity against the more recent A/Denver/1/1957 isolate (Fig. 4B). Furthermore, many serum samples collected from individuals born in the 1980s and 1990s had high antibody activity against this 1957 isolate.

There was no correlation between HAI titers against H3N2 viruses and H1N1 viruses (Fig. 4C). Approximately half of the people born between 1920 and 1979 had HAI antibodies against three contemporary H3N2 isolates tested. Younger people born in the 1980s and 1990s had higher numbers of H3N2 HAI positive samples (35–75%), with a decline in the number of positive samples from people born in the 2000s, although ∼55% of the samples were H3N2 positive. Few individuals had cross-reactive antibodies to A/Perth/16/2009 virus, which began circulating in late 2010.

### Relationships between Antibody Titers to Different Viruses

**Figure 5:**
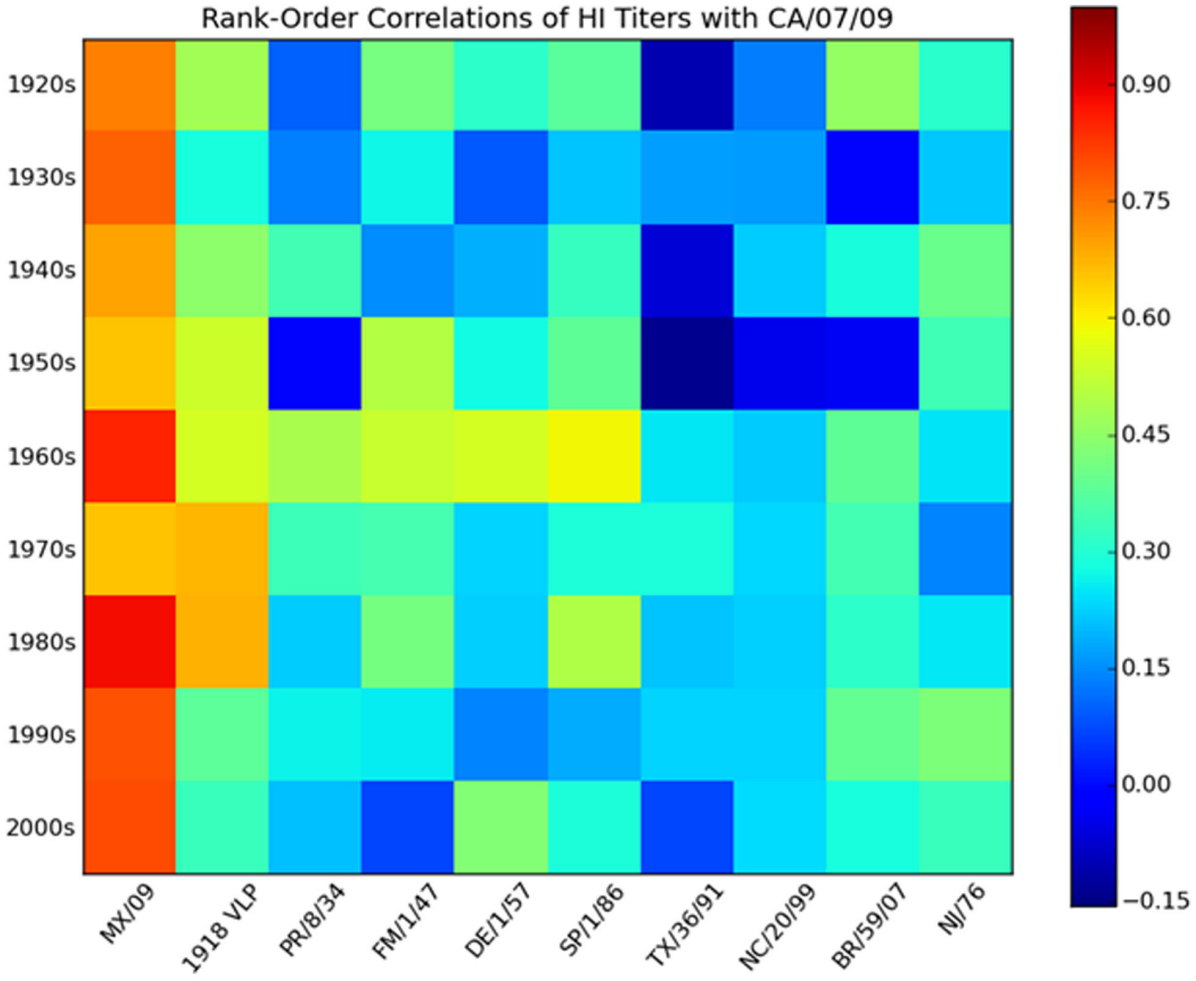
Correlations of CA/07/09 antibody titers with titers for other H1N1 isolates. The rank-order correlation coefficient between the CA/07/09 HAI titer and each other H1N1 titer is displayed for each decade of birth. As indicated at the right of the figure, warmer colors correspond to higher correlation coefficients.

To assess relationships between HAI reactivity against different viruses, we computed rank-order correlations of antibody titers for pairs of viruses in our panel. Correlations of the CA/07/09 titers with the HAI titers for the other H1N1 viruses are shown in Figure 5. For most age groups, the strongest correlation was to the other novel 2009 H1N1 virus, MX/4108/09 titer (for the 1970s the 1918 virus correlation is higher by a small amount). This is to be expected because the two 2009 viruses, though not identical, are very similar (two amino acid differences in the HA1 sequences). Not unexpectedly, the next highest correlation was to the HAI titer against the 1918 virus. However, for many age groups, the novel 2009/1918 HAI titer correlation is only moderate or weak, and for most cohorts, one or more seasonal isolates have comparable or stronger correlations. Among the weakest novel 2009/1918 HAI titer correlations were sera collected from individuals born in the 1990s (0.38) and 2000s (0.33), suggesting that a naïve human immune system does not produce strong cross-reactivity to the 1918 virus in response to the novel 2009 H1N1 virus. For all cohorts, the novel H1N1 2009 HAI titer correlates with titers of isolates separated from each other by decades of evolution. Correlations for all pairs of isolates are presented for each age cohort in the Fig. S1 and S2. These results exhibit additional moderate and strong correlations for pairs of isolates separated by significant antigenic drift. Many of these correlations are comparable to or stronger than the novel 2009/1918 HAI titer correlation for the same age group.

## Discussion

The novel 2009 H1N1 influenza virus emerged in March, 2009 and rapidly spread around the world resulting in the first influenza pandemic of the 21^st^ century. Unlike the 1957 or 1968 influenza pandemics, the 2009 pandemic was caused by a subtype of influenza (H1N1) that was already circulating in the human population. However, the sequence of the antigenically important hemagglutinin protein of the novel 2009 H1N1 virus was quite different from that of the circulating seasonal H1N1 strains, reflecting decades of independent evolution in the two lineages, since their divergence from a common ancestor. Thus, it might seem surprising that substantial pre-existing immunity to the novel H1N1 2009 virus was observed in older (though not necessarily elderly) adults, and that many such individuals carried pre-existing HAI antibodies that recognized the novel 2009 H1N1 virus. These observations raised questions about cross-reactivity of human antibodies and the relationship between the history of exposure to viral variants and the resulting profile of antibody reactivity.

Our group has measured the HAI reactivity of human sera to a panel of H1N1 isolates that includes the novel 2009 pandemic virus, the 1918 virus, and representative seasonal isolates from various eras ([9] and Fig. 3). Some aspects of these age-dependencies appear straightforward. Reactivity toward the 1918 virus is strongest in the elderly, as might be expected. HAI titers against the seasonal 2007 H1N1 isolate are highest for the young, who are the most likely infected with recent H1N1 isolates, and fairly low for other age groups. Titers against the 1986 isolate are low for those in the youngest cohort, who were born too late to be exposed to a similar virus, and antibodies to the 1947 isolate are highest at middle age groups and low in those born in the 1980s and later.

Other aspects of the pattern are more puzzling. Titers for the 1986 and 2007 isolates are low in those individuals born prior to each of those years, but high HAI titers against the 1991 and 1999 isolates are commonly found among older people. High titers for the 1947 virus can be found in sera collected from people born in the 1960s and 1970s, yet these same individuals have low HAI titers against the 1957 isolate (Fig. 3), which is similar to the post-1977 seasonal H1N1 that these individuals would have been exposed [18]. Furthermore, HAI titers for the 1957 isolate are high among those born in the 1980s and 1990s that are low HAI titers against the 1947 isolate.

**Figure 6:**
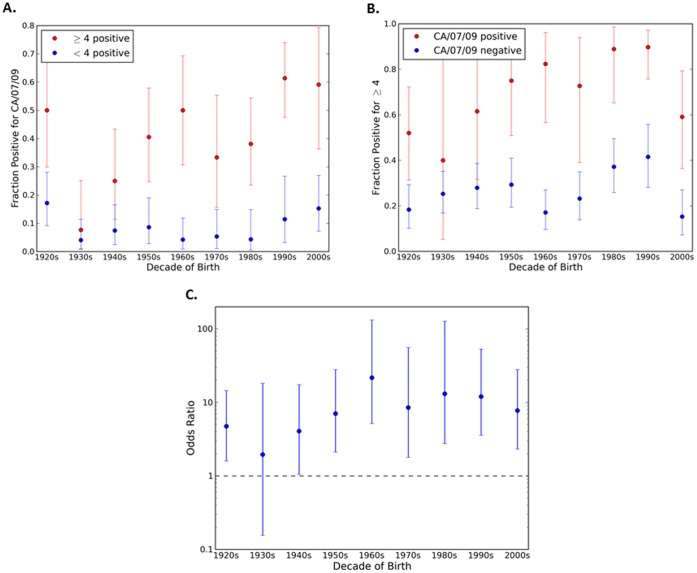
Seropositivity (HAI≥1∶40) for multiple seasonal H1N1 influenza viruses by decade of birth. Panel (A): The percentage of serum samples positive for A/California/07/2009 (novel H1N1) among those that were positive for ≥4 seasonal H1N1 (red) or positive for <4 seasonal H1N1 (blue). Panel (B): The percentage of serum samples positive for ≥4 seasonal H1N1 influenza viruses among those that were positive (red) or negative (blue) for A/California/07/2009 (novel H1N1) influenza. Panel (C): Odds ratio for seropositivity for A/California/07/2009 and seropositivity for ≥4 seasonal H1N1 influenza viruses. In all three panels the error bars indicate 95% confidence intervals.

There was a greater frequency of high HAI titers in the middle age groups compared to those individuals born in the 1920s, despite the somewhat higher frequency of HAI positive titers in the older age group. This may reflect the higher incidence of novel 2009 H1N1 virus infection in the middle age groups: positivity due to actual infection is likely associated with a higher HAI titer than positivity due to pre-existing antibodies. However, in addition to natural exposure, vaccination might contribute to this effect. Interestingly, approximately twice as many serum samples that were positive for novel 2009 H1N1 were more likely to be positive to ≥4 H1N1 tested in this study (Fig. 6). These factors, however, do not explain why high titers are more common in the intermediate groups than in the young. In fact, the higher rate of infection among the young makes this difference more difficult to explain. Presumably the few among the middle age groups who were infected developed a more potent antibody response than younger people who were infected, perhaps because of their greater prior exposure to influenza viruses. These observations hint at a complex relationship between viral exposures and antibody profile.

It has been proposed that pre-existing antibodies to the novel 2009 H1N1 virus in older adults can be explained by their exposure to 1918-like viruses in the early twentieth century [19]. Reports based on diverse lines of evidence have emphasized the similarity of the 1918 and novel 2009 HA molecules from the point of view of the immune system [10], [11], perhaps leaving the impression that they are practically equivalent. The results presented in this report indicate a more complex picture. HAI titers to the two antigens are undoubtedly positively associated (Figs. 2 and 5). However, the association is far from perfect, particularly for the older and younger cohorts. For most cohorts the relationship appears even less impressive when compared to the correlations between titers for the novel 2009 virus and later seasonal viruses (Fig. 5). Overall, comparison of the HAI reactivity against the HA antigens reveals additional moderate correlations between viruses that would not be considered antigenically close (Fig. S1 and S2). Many of these correlations are comparable to or higher than the novel 2009/1918 correlation by age group, further suggesting that the 1918 and novel 2009 hemagglutinin proteins should not be regarded as nearly equivalent. Also, only the very old would have been exposed to a close relative of the 1918 virus; whereas individuals in other age groups would only have experienced seasonal H1N1 viruses divergent from novel 2009 H1N1.

Both pre-existing antibodies to the novel 2009 H1N1 viruses and some puzzling patterns in the results presented here may reflect complex interactions between sequential exposures to different, but related, viruses. The antibody response to an influenza exposure may involve the activation of memory B cells that are present because of an earlier exposure to a somewhat different virus. Thus, earlier exposures can shape the antibody response to later exposures. One manifestation of this phenomenon is “original antigenic sin”: after an individual’s first exposure to a subtype, subsequent exposures to variants tend to elicit antibodies that react with the original virus, possibly at the expense of reactivity toward the novel variant [20], [21], [22], [23]. A beneficial consequence of the phenomenon may be that sequential exposures to drift variants lead to production of broadly protective antibodies. Therefore, MBCs with broader specificities will be disproportionately activated by drift variants and subsequent somatic mutation and selection may increase breadth. Since older individuals would have been exposed to a larger number of influenza variants, this might explain the age-dependent occurrence of pre-existing antibodies to novel H1N1 and the relationship between age and disease severity.

## Materials and Methods

### Viruses

Prototype strains chosen for this study were representative of antigenically distinct human seasonal influenza A H1N1 viruses in circulation during separate periods spanning nearly nine decades. Allantoic fluid antigens from influenza A/Brisbane/59/2007, A/New Caledonia/20/1999, A/Texas/36/1991, A/Singapore/6/1986, A/Denver/1/1957, A/Fort Monmouth/l/1947, A/Puerto Rico/8/1934, as well as the sequences based upon A/South Carolina/1/1918 isolate to generate virus-like particles (VLP) [9], [24]. Novel H1N1 isolates, A/California/7/2009 and A/Mexico/4108/2009, were used as representative isolates, as well as three H3N2 viruses, A/Wyoming/3/2003 and A/Sydney/5/1997. Influenza viruses were prepared by propagating virus in embryonated eggs as previously described.

### Sample Cohorts and Collections

Serum specimens used for this analysis have been previously described [14]. Briefly, serum samples were collected anonymously from extra laboratory specimens from mid-November to early December 2009. Anonymous blood samples were obtained from clinical laboratories and categorized by decade of birth from 1920–2009. Using hemagglutination-inhibition assays, approximately 100 samples per decade (n  = 846) were tested from blood samples drawn on hospital and clinic patients. University of Pittsburgh IRB approval [(exempt) #PRO09110164] was obtained. Blood samples were collected using the honest broker system at the University of Pittsburgh Medical Center and Children’s Hospital of Pittsburgh laboratories and given to investigators organized by decade of birth without other identifying information. The human serum samples were obtained from discarded blood collections at the following primary care medical visits. The samples had no identifying information, except decade of birth, and no other information was obtained. Each serum sample was tested in hemagglutination-inhibition assay (HAI) against two pandemic H1N1 (A/California/7/2009 and A/Mexico/4108/2009), as well as a panel of seasonal H1N1 isolates. Reference sera from individuals vaccinated with either inactivated trivalent seasonal Fluzone vaccine or pandemic H1N1 FluMist (GSK) vaccines were used as positive controls.

### Hemagglutination Inhibition Assay

The hemagglutination inhibition (HAI) assay was used to assess functional antibodies to the HA able to inhibit agglutination of turkey erythrocytes. The protocol was adapted from the CDC laboratory-based influenza surveillance manual as previously described [25]. To inactivate non-specific inhibitors, sera were treated with receptor destroying enzyme (RDE) prior to being tested [26], [27], [28], [29], [30]. The HAI titer was determined by the reciprocal dilution of the last well which contained non-agglutinated RBC. Positive and negative serum controls were included for each plate. HAI titers per group reflect appropriate estimates with 95% confidence intervals shown (by Clopper-Pearson method), along with plots showing odds ratios with 95% confidence limits (calculated using the “fisher.test” function of the R statistical package). For the correlations, Spearman’s rank-order correlation coefficient were calculated.

## Supporting Information

Figure S1 **Heat Map of antibody positivity by decade of birth for each H1N1 isolate listed.** Lighter colors indicate positive antibody titer per sample.
(TIF)

Figure S2 **Correlations of CA/07/09 antibody titers with titers for other H1N1 isolates.** The rank-order correlation coefficient between the CA/07/09 HAI titer and each other H1N1 titer is displayed for each decade of birth. As indicated at the right of the figure, warmer colors correspond to higher correlation coefficients.
(TIF)
