# Supplementary figures and images for: Complex Patterns of Human Antisera Reactivity to Novel 2009 H1N1 and Historical H1N1 Influenza Strains
Source: PLoS One. 2012 Jul 17;7(7):e39435. doi: 10.1371/journal.pone.0039435 (PMC3398940; doi:10.1371/journal.pone.0039435)

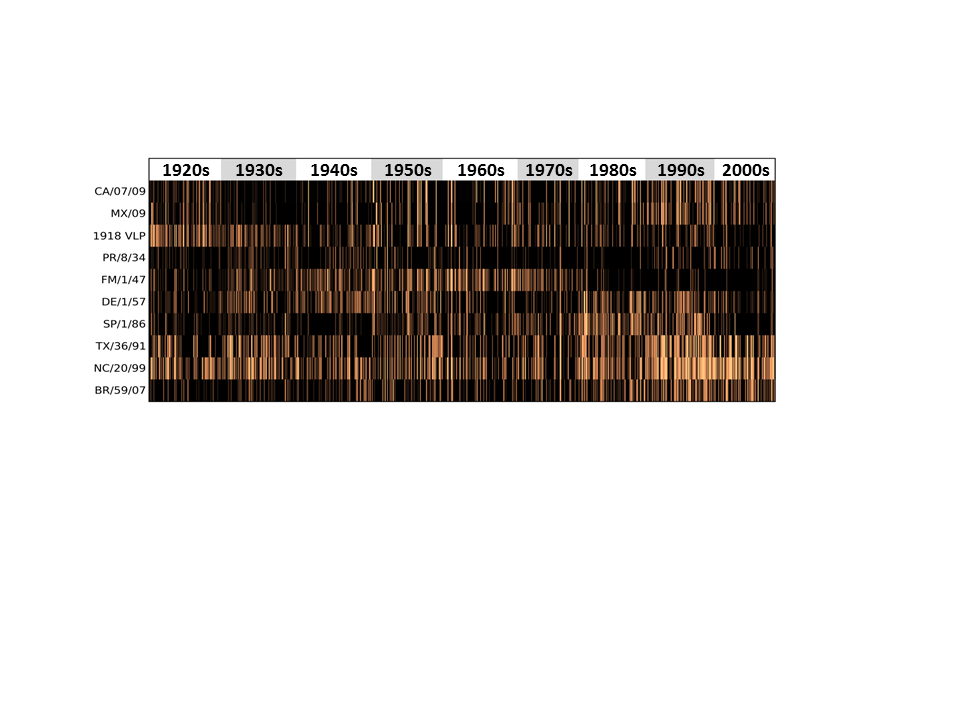

Supplement: Figure S1 — Heat Map of antibody positivity by decade of birth for each H1N1 isolate listed. Lighter colors indicate positive antibody titer per sample. (TIF) [file pone.0039435.s001.tif]

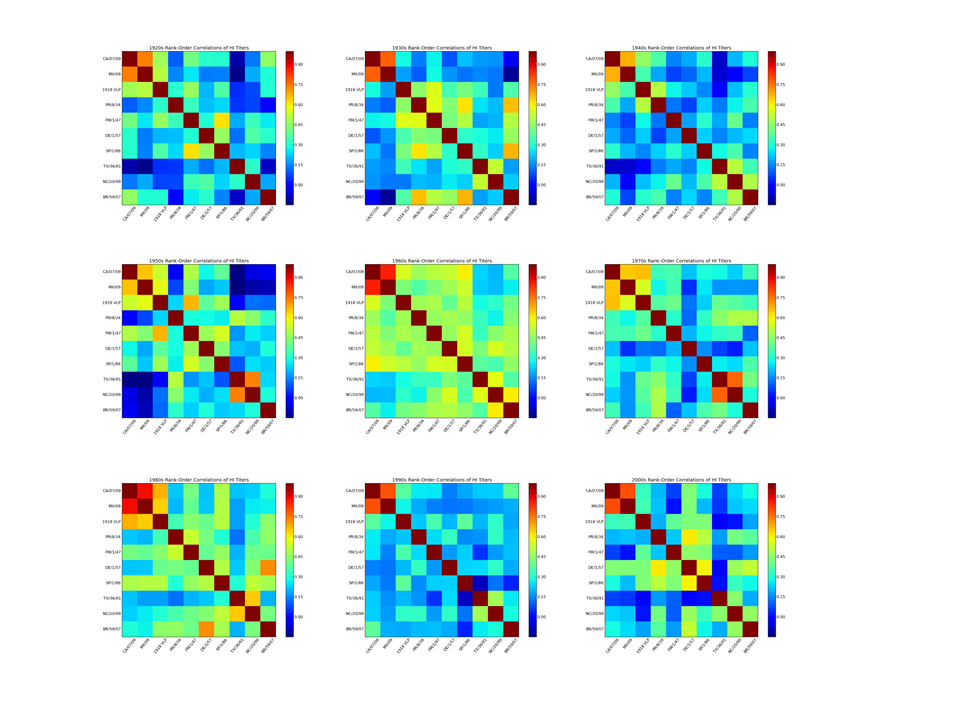

Supplement: Figure S2 — Correlations of CA/07/09 antibody titers with titers for other H1N1 isolates. The rank-order correlation coefficient between the CA/07/09 HAI titer and each other H1N1 titer is displayed for each decade of birth. As indicated at the right of the figure, warmer colors correspond to higher correlation coefficients. (TIF) [file pone.0039435.s002.tif]
